# Can We Improve Burnout in Neurology by Targeting Residency Program Resiliency?

**DOI:** 10.1212/NE9.0000000000200085

**Published:** 2023-07-06

**Authors:** Elizabeth A. Coon, Colin P. West, Lyell K. Jones

**Affiliations:** From the Department of Neurology (E.A.C., L.K.J.), and Department of Internal Medicine (C.P.W.), Mayo Clinic, Rochester, MN.

Burnout is highly prevalent among neurologists and starts early, with nearly three quarters of neurology residents experiencing one or more symptoms of burnout.^1,2^ Resiliency is defined as a capacity to withstand or adapt to stressors in a way that generates growth.^3^ High resiliency is associated with lower burnout rates, yet the relationship is often framed to place the onus on the individual suffering from burnout. The problem of burnout is unlikely to be solely due to individual factors because physicians exhibit higher levels of resilience than the general working population of the United States.^3^ Beyond an individual level, resiliency can be ascribed to organizations, with highly resilient organizations responding effectively to crises and maintaining adaptability and agility while maintaining focus on their primary purpose and goals.^4^ This leads to the question: Can we promote resiliency at a residency program level to combat burnout in our future neurologists?

There has been little focus on resiliency at a residency program level in neurology. One study adapted resiliency concepts for academic medicine and proposed the following themes to promote organizational resiliency: culture, leadership, adaptive capacity, resources, learning, systems, and capital.^5^ This framework highlighted the interplay between organizational and individual resilience using themes of communication, sense of belonging, shared vision, and recognition of gifts. Ideally, resilient residency programs would model and teach organization-level resiliency themes to enable trainees to combat stressors in not only residency but also their careers. These skills could be considered additional instruments in a neurologists' tool box.

How do we encourage residency programs to model organizational resiliency? Studies of larger organizations may provide insights into best practices to conceive, measure, and strengthen organizational resiliency in residency programs.^4,5^ The first step we propose is to perform a qualitative research study of a nationwide community of residents and residency leaders using an appreciative inquiry approach to define shared themes of organizational resiliency. This would be followed by quantitative assessments to complement identified perspectives. These results would hone the definition of program-level resiliency in neurology education with a goal of obtaining consensus on what measures would best evaluate and subsequently support improvements in resiliency at a training program level.

Identified themes from this organizational resilience framework could then be extended and adapted to the unique needs and environments of individual residency training programs. For example, leadership's use of a shared program vision could foster a sense of belonging to nurture an individual's resilience. A resilient program would be adaptable and agile in managing change while continually improving and maintaining clear communication. Program-level resiliency efforts should be integrated with the continued focus on well-being of individuals in the program. Barriers to creating a resilient program would need to be addressed and the work of fostering resiliency reconciled with competing priorities.

Considering resilience as both an individual and training program characteristic would advance medical education toward a critically important goal: creating resilient residency programs that foster sustainable learning and working environments to enable all learners to combat burnout and ultimately thrive in training and their careers.

## Appendix Authors

**Table:**

Name	Location	Contribution
Elizabeth A. Coon, MD	Department of Neurology, Mayo Clinic, Rochester, MN	Drafting/revision of the manuscript for content, including medical writing for content; study concept or design
Colin P. West, MD, PhD	Department of Internal Medicine, Mayo Clinic, Rochester, MN	Drafting/revision of the manuscript for content, including medical writing for content; study concept or design
Lyell K. Jones, MD	Department of Neurology, Mayo Clinic, Rochester, MN	Drafting/revision of the manuscript for content, including medical writing for content; study concept or design
